# The Scraping out (Curetting) of Chancroid

**Published:** 1890-08

**Authors:** O. Peterson

**Affiliations:** Syphilo-Derm tological Society of St. Petersburg


### THE SCRAPING OUT (CURETTING) OF CHANCROID.*

PAPER BY DR. O. PETERSON,

Before the Syphilo-Dermatological Society of St. Petersburg.

---

Among the numerous means employed against the spread of venereal diseases, the quickest possible cure of the individual patient, hence the reduction of the number of infection-bearing cases, plays a not unimportant role. Consequently, we take upon ourselves the task of improving, as far as possible, existing methods of treating these diseases. As is known, much has already been done in this field, but much yet remains to be done.

Favor me to-day by directing your attention to the question of treatment of chancroid. Although this must be considered wholly in the class of "milder" venereal diseases, yet it possesses an importance not to be underestimated—in view of the possible occurrence of bubo, which may render the patient unfit for work for weeks and months; while the quickest possible healing of the chancroid is capable of removing the likelihood of bubo appearing.

In describing the treatment of chancroid, Prof. Gay† calls attention to three points which should never be lost sight of, viz.:

*Translation from Monatshefte für Praktische Dermatologie, Band x., No. 9.

†Cursus der Venerischen Krankheiten. 3 Ansgabe, 1888, pag. 217 (russ.).

1. The quickest attainment of cleanliness of the ulcer.

2. The quickest possible protection of the vicinity from infection with the secretion.

3. The quickest limitation of the inflammation, thus retarding the spreading of the ulcer.

Altogether one can offer no objections to these propositions, and they accord essentially with what I shall have to say.

In order to treat a disease rationally, is it essential to know its etiology? We know that this disease is purely local, contagious, and in the highest degree capable of infecting. The question as to whether the infecting agent is of chemical nature (a ptomaine), or a micro-organism, remains as yet unanswered. As is well-known to you, a work by Finger recently appeared in which he seeks to show that a true chancroidal virus does not exist. According to Finger*, chancroid is simply a product of pus transferrence; any pus can, under favorable circumstance, produce, upon man, ulcers from which inoculations can be further successfully made. But he confesses, some lines further, that these "favorable conditions" are unknown to us. If Finger's conclusions are correct, we must wonder why no chancroids are observed in the surgical clinics, where one comes in contact with so many different kinds of pus.

For a long time the authors who have interested themselves in this question have sought the infecting agent among micro-organisms. The last work upon this subject, by the Italian physician, Primo-Ferrari, discloses an interesting fact. Ferrari† (in 1885) reported the discovery of bacilli in the epithelial cells and pus corpuscles which he had removed from previously sterilized chancroidal ulcers. These were much thinner and smaller than the lepra and "tubercle" bacilli. With the view of testing Ferrari's statements, I made preparations of chancroid secretions and found, generally, three kinds of bacilli and two of cocci. My observations are not yet concluded, and I have not made "pure cultures," but I am sure I have constantly found Ferrari's "rodlets" among the micro-organisms observed; also, the other

*Die Syphilis und die Venerischen Krankheiten. 1880. p. 103. (Not very recent. Trans.)

†Jullien, Traite pratique des maladies veneriennes. Paris, 1886.

organisms progressively decreased in number while the "Ferrari bacilli" increased—in the transparent secretion from the original vesicle of chancroids produced by further inoculation.

I am personally convinced from this that we must seek the infecting virus of chancroid among micro-organisms, and hence the chief indicatioin in the treatment is to destroy this organism by disinfecting the ulcer, this indeed being already partially attained through various methods of treatment.

A long list of remedies are offered in text-books, "one suggesting this and another that" (treatment). Manssurow* divides these remedies into two groups, the abortive and the curative. Jullien designates a "caustic," a "disinfecting," and an "astringent" method.

For abortive treatment, authors in general strongly recommend such caustic agents as Vienna paste, sulphur (?) caustic potash, the actual cautery (incandescent), and the Paquelin cautery. Prof. Gay says, with full justice, that these agents can only be employed in the first four days, since they produce great pain, caustic potash and Vienna paste tending to undesirable destruction of substance. It is indeed full time that they be stricken from the regulation treatment of chancroid! As concerns cauterization with nitrate of silver in substance, I feel it my duty to add that it usually produces such a degree of inflammatory induration that it is difficult to differentiate such a (badly treated) chancroid from a chancre.

Professor Gay further quotes the following measures as frequently employed:

1. Compression with nitrate of silver solution (1-3 ag. nit. to 300 aq. distill.); sulphate of copper, 1-600, or 1-300; chloride of zinc, 1-200; or, if the course is slower, carbolic acid 1-2 to 30; aromatic waters, tannin lotions, etc.

2. Powdering, formerly with calomel, now with iodoform or iodol.

3. Salves—of which, however, Prof. Gay disapproves, decidedly, and, as I think, with perfect justice.

In Manssurow's work we also find such remedies as "aqua

*Cursus der Venerischen Krankheiten. Moscow, 1888.

phagedenica nigra," and acid carbolic 1, to olive oil 600. He believes compression with chloride of zinc solution (1-600) the best measure. Further, he mentions salves, as ungt. basilic nigrum, *i. e.*, a tar ointment, and a salve composed of salicylic acid, 4 parts to simple ointment 30 parts, etc. Finger advises dressings with solutions of sulphate of copper (1-100), or caustic potash 1-90; ointments of red precipitate of mercury 1 part to vaseline 90 parts, etc. Dressings, with any of the above applications, to be made twice per day.

Did I not fear to abuse your attention, I might yet mention a great number of remedies. However, I will only call attention to one other remedy (in Russian literature) which is not mentioned in the text-books, viz., "phenol-trichloride," proposed by Dr. Rubez, but the observations of Tomaschewski, in Prof. Tarnowski's clinic, have demonstrated the complete inapplicability of this drug in chancroid. The greater number of the remedies just described have been employed, during the past ten years, in my department in the Alexander Hospital, iodoform having, however, been, and is, most used, sometimes in powder, again in ethereal solution. Experiments were made, besides, with naphthol (1881), salicylic acid (1882), bismuth subnitrate and creta alba in 1883. In recent chancroids these remedies were effective as disinfectants—excepting the chalk. But naphthol irritated and the salicylic acid formed dough-like nodules, beneath which pus collected.

Inspired with the wish to heal chancroids with all possible haste, I finally chose three methods, which have given me good results:

1. Were it desirable to heal the ulcer as soon as possible, just as in follicular chancre, which is always of long duration, I fixed the lesion with a pincette and clipped it away with a stroke of the scissors, including a portion of the adjacent skin. Edges of wound were stitched together, and "first intention" was usually obtained in three days. It is self-evident that the operation must be done under full antiseptic precautions. It may be said against excision that it is painful and impracticable in such a position as the glans penis. On the other hand, I may declare myself unable to agree with Prof. Gay when he says, "practice has proven the complete impracticability of this method." He founded this view upon the cases in which several ulcers were present, in which the operation would lead to great deformity, as one could not cut around the extreme border of the ulcer. I repeat that one cannot employ this method in *every* case.

2. Painting with tincture of iodine. This method gives good results, for example, in recent (or in older, indolent) ulcers, but, even here, there sometimes occur symptoms of irritation.

3. "Scraping out." I have now employed this method a year and a half, and have obtained such good results that I cannot too warmly recommend it to my colleagues. By scraping we change the chancroidal ulcer, in a few minutes, to a simple wounded surface (as I have often been able to demonstrate to my class, both in the clinic and the polyclinic), which healed, in shortest time, under the usual applications, as dusting with iodoform, or dressing with iodoform, or sublimate, gauze. The technique is extremely simple. After the ulcer is washed with a carbolic, or bichloride, solution and wiped with a piece of ether-soaked wool, take the smallest possible sharp spoon (curette) and scrape just as is customary in lupus, or an ordinary indolent ulcer, etc.

The method has a single disadvantage, viz., its painfulness; but this is easily obviated by injecting cocaine (solution) five or ten minutes before the operation. In this way the pain of "curetting" need scarcely be greater than that from cauterization with caustic potash, caustic paste, sulphur or nitric acid, remembering, besides, that all these produce crusts which remain fixed a longer or shorter time, and produce a not altogther mild inflammatory reaction.

It remains, now, only to demonstrate statistically how the results which I have attained in my department, by this method, were established. In order not to weary you with "case-histories," I will only mention that the average time of healing in three hundred curettings was 9 9-10 days (under former methods, 1887, the uncomplicated chancroids required an average of 19 9-10 days, and in the year 1880 fully 25 2-10 days). Twenty-six times healing occurred in three days, twenty times in five days, and so on. In single cases (generally of anæmia or chronic alcoholism) the healing required twenty-two to twenty-five days, but under every other method mal-nutrition likewise retarded recovery.

In conclusion, I add a table of "days of duration," which will make clear the results of methods of treatment in the Alexander Hospital during the past eight years. It is clear that the length of time in the hospital is proportional to the number of complicated chancroids.

There were three thousand and fifty-five (3,055) cases of chancroid treated in Alexander hospital in the years 1881 to to 1885 (inclusive).

| Years | 1881 | 1882 | 1883 | 1884 | 1885 | 1886 | 1887 | 1888 |
|---|---|---|---|---|---|---|---|---|
| Number of chancroids | 279 | 401 | 364 | 410 | 289 | 513 | 697 | 102 |
| Percentage of complications | 68.8 | 51.8 | 56.9 | 70.3 | 54.0 | 51.5 | 50.5 | 62.1 |
| Average duration in days | 34.3 | 26.1 | 27.1 | 28.6 | 22.9 | 26.5 | 25.3 | 20.7 |

It is clear from this table that the average in days is proportional to the number of complicated cases; in the year 1888, however, in which curetting of the chancroid was systematically followed, the average duration, in "hospital days," is less than in other years, despite the fact that the number of complicated cases was greater than in the three preceding years—the lighter cases being treated as "out-patients." The average "in hospital days" in the first seven years (2,953 cases) was 27.5; after the introduction of "scraping out," 20.7 days—in other words, the duration of treatment was shortened about one week.

